# Emerging Trends and Thematic Evolution of Breast Cancer: Knowledge Mapping and Co-Word Analysis

**DOI:** 10.2196/26691

**Published:** 2021-10-28

**Authors:** Ali Sadatmoosavi, Oranus Tajedini, Omid Esmaeili, Firouzeh Abolhasani Zadeh, Mahdiyeh Khazaneha

**Affiliations:** 1 Department of Medical Library & Information Sciences Faculty of Management and Medical Information Sciences Kerman University of Medical Sciences Kerman Iran; 2 Department of Knowledge and Information Science Shahid Bahonar University of Kerman Kerman Iran; 3 School of Medicine Shahid Mohammadi Hospital Hormozgan University of Medical Sciences Hormozgan Iran; 4 Department of General Surgery School of Medicine, Afzalipour Hospital Kerman University of Medical Sciences Kerman Iran; 5 Department of Information Sciences and Medical Informatics Kerman University of Medical Sciences Kerman Iran

**Keywords:** scientometrics, breast cancer, co-word analysis, Scimat, science mapping

## Abstract

**Background:**

One of the requirements for scientists and researchers to enter any field of science is to have a comprehensive and accurate understanding of that discipline.

**Objective:**

This study aims to draw a science map, provide structural analysis, explore the evolution, and determine new trends in research articles published in the field of breast cancer.

**Methods:**

This study comprised a descriptive survey with a scientometric approach. Data were collected from MEDLINE using a search strategy based on Medical Subject Heading (MeSH) terms. This study used science mapping, which provides a visual representation and a longitudinal evolution of possible interrelations between scientific areas, documents, or authors, thus reflecting the cognitive architecture of science mapping. For this scientometric evaluation of the topic of breast cancer research, a very long period was considered for data collection. Moreover, due to the availability of numerous publications in the database, the assessment was divided into three different periods ranging from 1988 to 2020.

**Results:**

A total of 12,577 records related to scientometric studies were extracted. The field of breast cancer research demonstrated three diagrams containing the most relevant themes for the three chronological periods evaluated. Each diagram was plotted based on the centrality and density linked to each research topic. The research output in the field was observed to revolve around 8 areas or themes: *radiation injury*, *cardiovascular disease*, *fibroadenoma*, *antineoplastic agent*, *estrogen antagonistic*, *immunohistochemistry*, *soybean*, and *epitopes*, each represented with different colors.

**Conclusions:**

In the strategic diagrams, the themes were both well developed and important for the structuring of a research field. The first quadrant comprised motor themes of the specialty, which present strong centrality and high density (eg, corticosteroid antineoplastic age, stem cell, T-lymphocyte, protein tyrosine kinase, dietary, and phosphatidyl inositol-3-kinase). In the second quadrant of diagram, themes have well-developed internal ties but unimportant external ties, as they are of only marginal importance for the field. These themes are very specialized and peripheral (eg, DNA-binding). In the third quadrant, themes are both weakly developed and marginal. The themes in this quadrant have low density and centrality and mainly represent either emerging or declining themes (eg, ovarian neoplasm). Themes in the fourth quadrant of the strategic diagram are considered important for a research field but are not fully developed. This quadrant contains transversal and general, basic themes (eg, immunohistochemistry). Scientometric analysis of breast cancer research can be regarded as a roadmap for future research and policymaking for this important field.

## Introduction

One of the requirements for scientists and researchers to enter any field of science is to have a comprehensive and accurate understanding of that discipline [1]. Accordingly, knowledge of the concepts, history, framework, scope, components, and functions of each discipline of science, as well as analyzing and examining how these are linked in the intertwined chain of human sciences and demonstrating these links with the fields on which they are more dependent, is of key importance [2]. In general, this knowledge should facilitate the best assay to gain a comprehensive picture of the fields of activity and applications of that discipline, which should be used as a guide by those who have not yet determined their future research passageway [3].

Science mapping is the analysis of publications within a scientific field from different viewpoints; it helps visualize a general assessment of a given field [4]. By using this map, the course of changes and developments in the field can be plotted to differentiate the fields with the most and the least proximity. Science mapping is undertaken to identify points of knowledge that follow “hot topics” and current trends in a given field [5]. A science map drawn based on the scientific research outputs of a field makes it possible to study the emergence of new fields and the cessation of some saturated scientific fields [6,7]. Simply put, the purpose of a science map is to depict the results of the analysis of publications of a scientific field from different angles and to provide an overview of that field [8]. Science maps attempt to showcase the processes of growth, integration, and disintegration of different fields of science over time. Scientific domains in these maps are determined in proportion to the level of activity of scientists, and the empty spaces in the illustrative map indicate unworked or unknown domains of science. This illustration thus showcases the growth, integration, or disintegration of different scientific fields over time [9,10]. In recent times, scientometrics—as a branch of information science and a bibliometric subfield—has been used in a plethora of studies to quantitatively examine emerging research patterns in the literature [11].

One of the most widely used methods for analyzing the structure of knowledge in various fields and drawing science maps is *co-word analysis* that examines the co-occurrence of keywords in the title, abstract, or text of articles. Therefore, co-word analysis is done on a set of published articles in a specific subject area [12]. By analyzing keywords used in articles of a specific research field, we can better understand the content of the common topics in that field [13,14]. Moreover, by measuring the relative intensity of these co-occurrences, simplified representations of concept networks in a given field can be illustrated [15].

Co-word analysis can reveal the main topics of the field under study, semantic structures, and the evolution of those works over time. In a co-word analysis, it is assumed that the most frequent words have a greater impact in a field of study than words that appear less frequently. Moreover, co-word analysis allows us to reveal emerging trends and changes in paradigms to facilitate predicting the direction of future research [13]. Co-word analysis can be used as a powerful tool to enable the follow-up of structural changes and the development of the sociocognitive network. This method also helps us identify emerging topics in scientific fields and draw a clear path for future research [16]. Furthermore, these networks were mapped by running network analyses using cosine link reduction and pathfinder networking scaling techniques [17].

One of the most important topics in medical research is breast cancer. Breast cancer is the most frequent type of cancer among women, affecting approximately 2.1 million women each year. It also causes the highest number of cancer-related deaths among women [18]. A total of 268,600 new cases of invasive breast cancer were estimated to be diagnosed among women and approximately 2670 cases among men in the year 2019 [19]. In addition, an estimated 48,100 cases of ductal carcinoma in situ were estimated to be diagnosed among women. Approximately 41,760 women and 500 men were expected to die from breast cancer in 2019 [20,21].

As in other fields of science, new research studies continually emerge in the field of breast cancer, leading to advancements in the field. Many of these studies often have some similarities and overlaps. For a variety of reasons, the volume of research suddenly sees a surge in some subfields, and with such increments, thematic overlaps can occur. However, in other areas, little research may be done over months and years. Given the importance of research in the field of medicine, in general, and breast cancer, in particular, it is necessary to provide a broad picture of the status of research conducted in this field. In other words, the structure of knowledge in this field should be revealed using techniques such as co-word analysis to demonstrate how this field has developed over time, and more importantly, to better understand the emerging topics, issues, and themes that have developed in this field.

This study uses co-word analysis to examine articles published in the field of breast cancer and improve or continue the necessary context for correction, continuation, or promotion of the pattern of their scientific behavior by gaining an understanding of the interests and tendencies of researchers in the field over time. Accordingly, this study aims to draw a science map, provide structural analysis, explore the evolution, and find new trends in articles published in the field of breast cancer by addressing the following research questions:

1. What are the most important research areas in the field of breast cancer?
2. Under which of the 4 themes (ie, motor themes, specialized and peripheral themes, emerging or disappearing themes, and general and basic themes) are breast cancer thematic areas classified in the strategic diagram?
3. What are the most important issues in terms of frequency and intensity?
4. How have breast cancer thematic areas been developed across different periods?

## Methods

### Search Strategy

We used the MEDLINE database to retrieve and extract bibliographic information from breast cancer-related research articles. MEDLINE is the premier bibliographic database of the US National Library of Medicine (NLM) that contains more than 25 million references to journal articles in the field of life sciences with a concentration on biomedicine [22]. A distinctive feature of MEDLINE is that the records are indexed with NLM Medical Subject Headings (MeSH) [23,24]. With regard to subject areas, MEDLINE includes biomedicine and health care research, broadly defined to encompass those areas of life sciences, behavioral sciences, chemical sciences, and bioengineering that are needed by health professionals and others engaged in basic research and clinical care, public health, health policy development, or related educational activities. MEDLINE also covers life sciences fields vital to biomedical practitioners, researchers, and educators, including aspects of biology, environmental science, marine biology, plant and animal science, as well as biophysics and chemistry [24].

To further validate the retrieved results, the search strategy used in this study was limited to research papers published in core clinical journals. The period covered in this study included all the years covered by this database (from 1950 to March 24, 2020). In other words, this study evaluated a total sample of 12,577 research articles published across 70 years. The retrieved records were saved as full records in plain text using tab-delimited and RIS (research information systems) formats. Finally, after saving the retried data, the related files were integrated and saved as a single file for subsequent use.

### Data Acquisition and Processing

#### Overview

This study has been written on the basis of co-word analysis. Bibliometric methods explore the impact of a research field, a group of researchers, or a particular paper [25]. In this study, we used science mapping, which provides a visual representation and a longitudinal evolution of the interrelations between scientific areas, documents, or authors, reflecting the cognitive architecture of science mapping [26].

We used SciMAT [22,27], which is a powerful open-source science mapping [28] software. The tool allowed us to analyze the evolution and relevance of the literature focused on breast cancer. This tool was designed according to the science mapping analysis approach, which allows researchers to analyze a research field; detect and visualize its conceptual subdomains (particular topics or themes or general thematic areas); and develop a longitudinal framework to analyze and track the conceptual, intellectual, or social evolutions of e-government through the course of consecutive periods [29]. Different bibliometric tools are available to perform this kind of study [29], but SciMAT has some characteristics that distinguish it from other science mapping analysis tools.

SciMAT divides the analysis into four phases. A detailed explanation of these phases can be found elsewhere [7,30], although a brief description is provided below.

#### Detection of Research Themes

The first phase involves detection of research themes. This phase summarizes the first five steps of the workflow of science mapping analysis. In each period studied, the corresponding research themes are detected by applying a co-word analysis [31] to the raw data of from the published documents in the research field, followed by clustering of keywords to topics or themes by using the simple centers algorithm [32]. Formally, the methodological foundation of co-word analysis is based on the idea that the co-occurrence of keywords describes the content of the documents in a corpus [33]. These co-occurrences of keywords can be used to build co-word networks [34], and these networks can be associated with research themes using clustering tools. The co-occurrence frequency of two keywords is extracted from the corpus by counting the number of documents in which the two keywords appear together. Once the co-word network is built, each arc or edge will have in its weight the co-occurrence value of the linked terms. Next, the weight of each edge is transformed to normalize it (extracting the similarity relations between terms) using their keyword and co-occurrence frequencies [35].

#### Data Visualization

In this phase, the detected research themes are visualized using two different visualization instruments: a strategic diagram [22,36-38] and a thematic network. Each theme can be characterized by two measures [12,13]—centrality and density.

**Figure 1:**
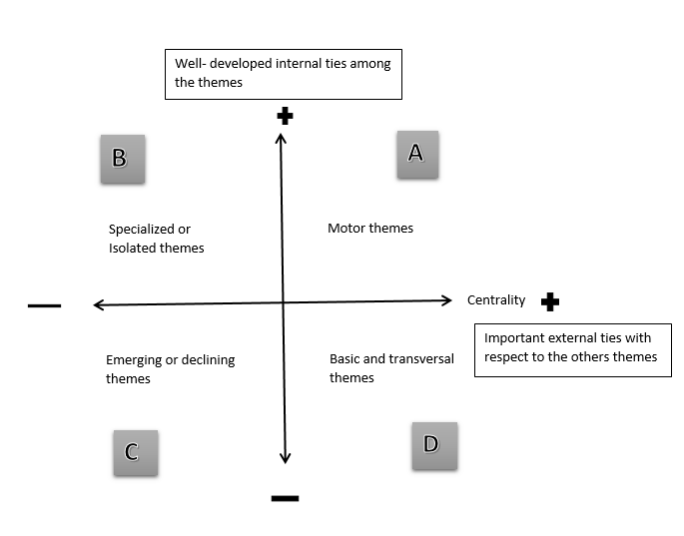
Strategic diagram in 2D and the 4-group classification: (A) motor themes, (B) specialized or isolated themes, (C) emerging or declining themes, (D) basic and transversal themes.

Centrality measures the degree of interaction of a network with other networks and shows the strength of external ties to other themes [39]. This value can be considered as the measure of the importance of a theme in the development of the entire research field analyzed. Density measures the internal strength of the network and reflects the strength of internal ties among all the keywords that describe the research theme. This value can be considered as a measure of the theme’s development [40]. Once the centrality and density rankings are calculated, the themes can be laid out in a strategic diagram. Given both measurements, a research field can be visualized as a set of research themes, mapped in a 2D strategic diagram (Figure 1), and classified into the following four groups:

1. Motor themes
  that are both well developed and important for the structuring of a research field; these themes present strong centrality and high density.
2. Specialized and peripheral themes
  that have well-developed internal ties but unimportant external ties, as they are of only marginal importance for the field.
3. Emerging or declining themes
  that are both weakly developed and marginal; the themes in this quadrant have low density and low centrality.
4. Basic and transversal themes
  that are important for a research field but are not developed (eg, notes in computer science) [26].

#### Discovery of Thematic Areas

**Figure 2:**
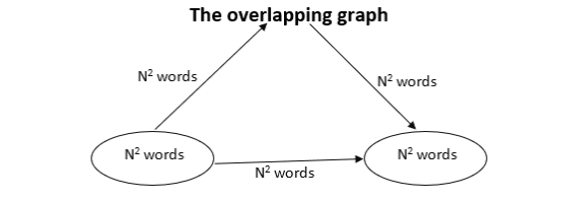
The overlapping graph. The horizontal arrow represents the number of items shared by both periods. The upper incoming arrow represents the number of new items in the second period, and the upper outgoing arrow represents the items that are presented in the first period, but not in the second period [28,41].

**Figure 3:**
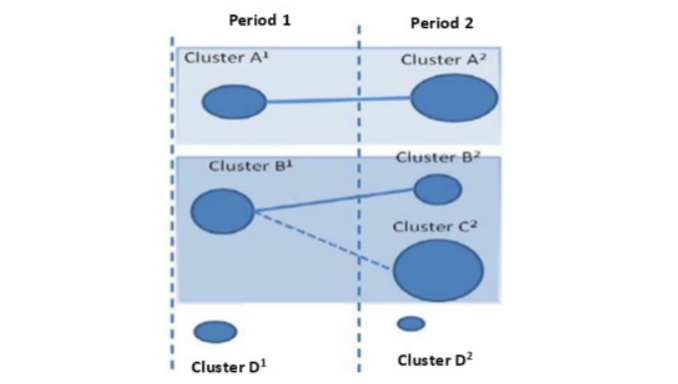
The evolution map: cluster D1 is discontinued, and cluster D2 is considered to be a new cluster.

The next phase involved temporal or longitudinal analysis. In this phase, the evolution of the research themes over a set of periods is first detected and then analyzed to identify the main general areas of evolution in the research field, their origins, and their interrelationships. This allows the discovery of the conceptual, social, or intellectual evolution of the field. SciMAT can build an evolution map [29] and an overlapping items graph (Figure 2) [41] to detect the evolution areas (see Figure 3).

For this purpose, an inclusion index is used to detect conceptual nexuses between research themes in different periods and thus identify the thematic areas in a research field. In addition, as each theme is associated with a set of documents, each thematic area can also have an associated collection of documents, obtained by combining the documents associated with its set of themes. Thus, the evolution map shows the temporal evolution of research themes of e-government, and the overlapping graph represents the number of associated keywords (Figure 2) [42].

#### Performance Analysis

In this phase, the relative contribution of research themes and thematic areas to the whole research field is measured (quantitatively and qualitatively) and used to establish the most prominent, most productive, and highest impact subfields. This performance analysis is developed as a complement to the analysis step of the science mapping workflow. Some bibliometric indicators in this phase used include the number of published documents, number of citations, and the different types of h index [43,44].

Eventually, three diagrams were represented based on the three temporal visualization phases. Following the science mapping workflow, visualization techniques were used to represent a science map and the results of different analyses. In this sense, the network results from the mapping step were represented in the form a strategic map, evolution map, and overlapping graph. Finally, when the science mapping analysis was completed, experts analyzed the results and maps, using their experience and knowledge.

### Ethics Approval

Since this was a metadata analysis of published work, approval from an ethics committee was not required.

## Results

### Overview

After retrieving a total of 12,577 records related to scientometric research, the importance of keywords (Table 1) and various journals was demonstrated (Table 2).

**Table 1:** Most frequently used terms in the selected articles (N=12,577).

Sr. no.	Source titles	Records, n (%)
1	Breast neoplasms	11,855 (97.9)
2	Prognosis	1919 (15.8)
3	Lymphatic metastasis	1735 (14.3)
4	Mastectomy	1520 (12.6)
5	Mammography	1435 (11.8)
6	Neoplasm staging	1420 (11.7)
7	Neoplasm recurrence local	1208 (10)
8	Neoplasm metastasis	1154 (9.5)
9	Risk factors	1115 (9.2)
10	Estrogen receptors	1113 (9.2)
11	Time factors	1014 (8.4)
12	Age factors	955 (7.9)
13	Carcinoma	889 (7.3)
14	Carcinoma ductal breast	814 (6.7)
15	Combined modality therapy	781 (6.4)
16	Axilla	779 (6.4)
17	Carcinoma intraductal noninfiltrating	753 (6.2)
18	Antineoplastic combined chemotherapy protocols	720 (5.9)
19	Lymph nodes	686 (5.7)
20	Mass screening	677 (5.6)
21	Antineoplastic agents	674 (5.6)
22	Biomarker tumor	655 (5.4)
23	Immunohistochemistry	640 (5.3)
24	Chemotherapy adjuvant	594 (4.9)
25	Neoplasm invasiveness	591 (4.9)
26	Lymph node excision	590 (4.9)
27	Tamoxifen	581 (4.8)
28	Receptor progesterone	563 (4.6)
29	Mastectomy segmental	543 (4.5)
30	Menopause	526 (4.3)

**Table 2:** The top 30 journals with the highest number of articles published on breast cancer (N=12,577).

Row	Source title	Records, n (%)
1	Cancer	3321 (27.4)
2	Lancet London England	567 (4.7)
3	American Journal of Surgery	523 (4.3)
4	British Journal of Surgery	454 (3.7)
5	Radiology	453 (3.7)
6	Journal of Clinical Pathology	376 (3.2)
7	American Journal of Roentgenology	347 (2.9)
8	The New England Journal of Medicine	331 (2.7)
9	JAMA^a^	330 (2.7)
10	American Journal of Clinical Pathology	320 (2.6)
11	Annals of Surgery	295 (2.4)
12	The American Journal of Pathology	289 (2.4)
13	Medicine	256 (2.1)
14	Archives of Surgery (Chicago, Illinois) 1960	232 (1.9)
15	Archives of Pathology Laboratory Medicine	199 (1.6)
16	British Journal of Radiology	199 (1.6)
17	Endocrinology	194 (1.6)
18	Surgery	193 (1.6)
19	British Medical Journal (Clinical Research Ed.)	192 (1.6)
20	Surgery Gynecology Obstetrics	186 (1.5)
21	Journal of the American College of Surgeons	177 (1.5)
22	Journal of Clinical Endocrinology and Metabolism	153 (1.3)
23	Plastic and Reconstructive Surgery	152 (1.3)
24	British Medical Journal	145 (1.2)
25	Journal of Clinical Investigation	126 (1)
26	Southern Medical Journal	118 (1)
27	American Journal of Public Health	106 (0.9)
28	Annals of Internal Medicine	102 (0.8)
29	Surgical Clinics of North America	96 (0.8)
30	The American Journal of Clinical Nutrition	94 (0.8)

^a^JAMA: Journal of the American Medical Association.

### Result for the Thematic Period (1987-2020)

**Figure 4:**
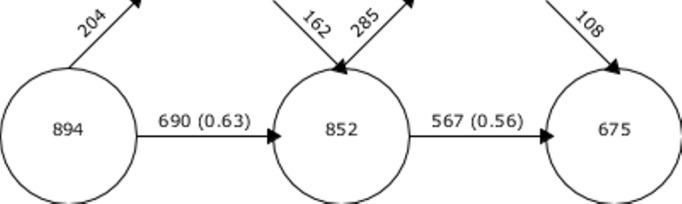
Thematic areas in the three evaluation periods based on centrality and density. The horizontal output arrows represent the number of concepts that served as the input for the next period; the vertical output arrows represent the number of concepts that exited a given period and were considered less important; and the vertical input arrow represents the number of concepts that received attention. In the second period, 852 new concepts appeared in the articles and 690 concepts were considered from the previous period. Of these 567 concepts entered the third evaluation period, and 675 new concepts appeared in the articles.

Figure 4 shows the number of concepts related to the thematic area of breast cancer in the three 11-year periods spanning from 1988 to March 31, 2020.

In the first period, the highest centrality was found for immunohistochemistry (IHC), and the highest density was related to the soybean theme. In the second period, the highest centrality was found in the antineoplastic themes, and the highest density was detected for the themes isoflavones and enzyme inhibitors. In the third period, the highest centrality was found for the antineoplastic agent theme, and the highest density was detected for the vegetable theme.

The strategic diagram of breast cancer was drawn based on the abundance of articles in the 4 thematic areas, including motor cluster, basic and transversal cluster, highly developed cluster, and emerging and declining cluster. The most important topics were found in the motor cluster, which are displayed in 10-year periods.

### First Evaluation Period (1989-1998)

**Figure 5:**
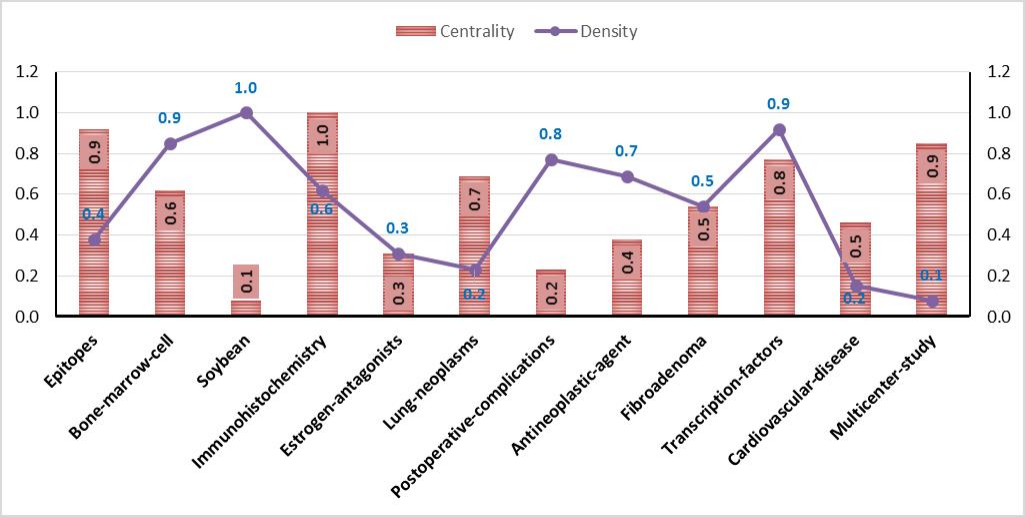
Breast cancer–related concepts identified in the first evaluation period based on density and centrality from 1988 to 1998. Date adopted from global statistics retrieved from the Web of Sciences.

In the first period, the upper-right quadrant (ie, motor cluster) comprised transcription factors, bone marrow cell, immunohistochemistry, and fibroadenoma, indicating the important role of these concepts in the field of breast cancer from 1989 to 1998 (see Figure 5). A transcription factor is a protein that controls the rate of transcription by binding to a specific DNA sequence. Immunohistochemistry is one of the best ways to detect these factors (Multimedia Appendix 1).

### Second Evaluation Period (1999-2009)

**Figure 6:**
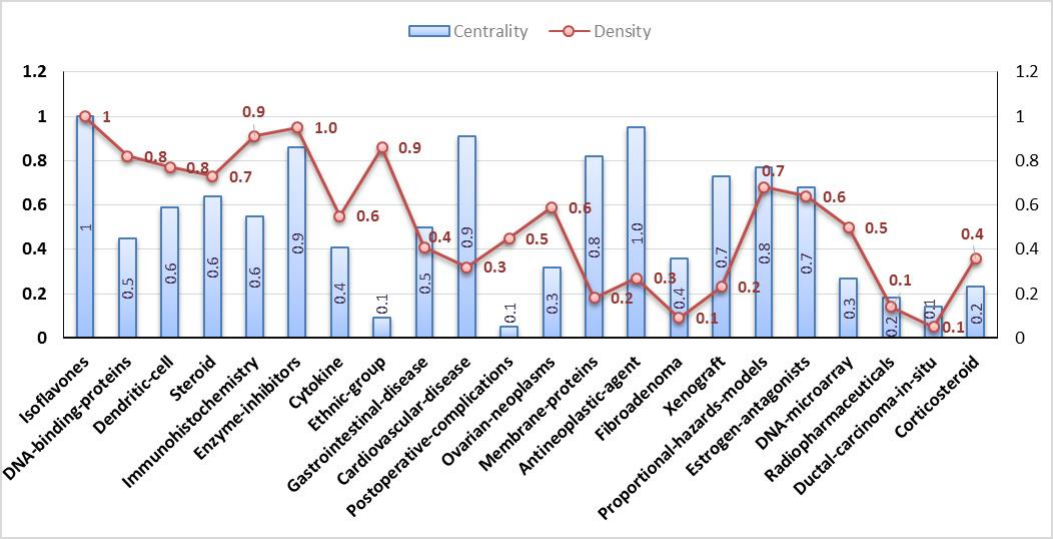
Breast cancer–related concepts identified in the second evaluation period based on density and centrality from 1999 to 2009. Date adopted from global statistics retrieved from the Web of Sciences.

The concepts related to the motor theme included isoflavones, enzyme inhibitors, immunohistochemistry, estrogen, proportional hazard model, and steroid. Soy isoflavones are enzyme inhibitors similar to lipoxygenase. Moreover, there is a close relation between suppression of dendritic cell maturation and functions by isoflavones (phytoestrogen; see Figure 6). Therefore, soy isoflavones can bind to estrogen receptors and act as an estrogen antagonist (Multimedia Appendix 2).

### Third Evaluation Period (2010-2020)

**Figure 7:**
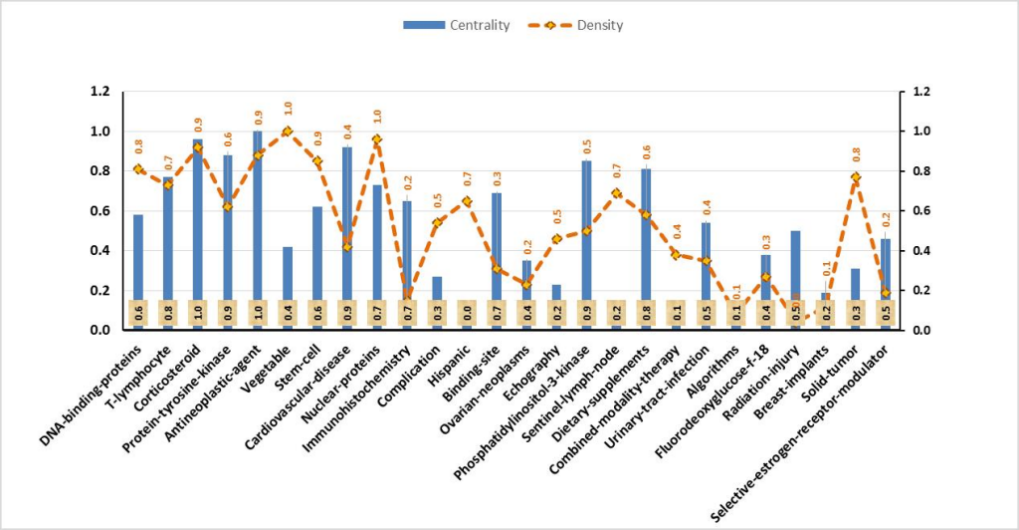
Breast cancer–related concepts identified in the third evaluation period based on density and centrality from 2010 to 2020. Date adopted from global statistics retrieved from the Web of Sciences.

The concepts of the motor theme in the third evaluation period included revealing corticosteroid antineoplastic age, stem cell, T-lymphocyte, protein tyrosine kinase, dietary, and phosphatidylinositol-3-kinase, indicating the importance of these topics in this period (Figure 7).

Steroids are important biodynamic agents and can be used as a particular agent for receptor-mediated diseases just like for breast cancer. Furthermore, infiltrative T-lymphocytes are related to invasive breast cancer.

Protein tyrosine phosphatases have a crucial role in the regulation of stem cell renewal and differentiation. Some studies have shown relations between DNA-binding protein oxidation and dietary supplements that contain plant extracts and vitamins (Multimedia Appendix 3).

## Discussion

### Principal Findings

After retrieving a total of 12,577 records related to scientometric studies, we were able to demonstrate important keywords, including *breast neoplasms*, *prognosis*, *lymphatic*, *metastasis*, *mastectomy*, *mammography*, and *neoplasm staging*. Indeed, examples of 30 journals with the highest number of articles published on breast cancer were *Cancer*, *Lancet (London, England)*, *The American Journal of Surgery*, *British Journal of Surgery*, *Radiology*, *Journal of Clinical Pathology*, *American Journal of Roentgenology*, *The New England Journal of Medicine*, *JAMA (Journal of the American Medical Association)*, and *The* *American Journal of Clinical Pathology*. Moreover, across the three evaluation periods, the themes with the highest density and centrality were observed in the first period, with the highest centrality was related to immunohistochemistry and the highest density related to the soybean theme. In the second evaluation period, the highest centrality was associated with the antineoplastic themes, and the highest density was observed in the isoflavones and enzyme inhibitor themes. In the third evaluation period, the highest centrality was related to the antineoplastic agent theme, and the highest density was observed in the vegetable theme.

**Figure 8:**
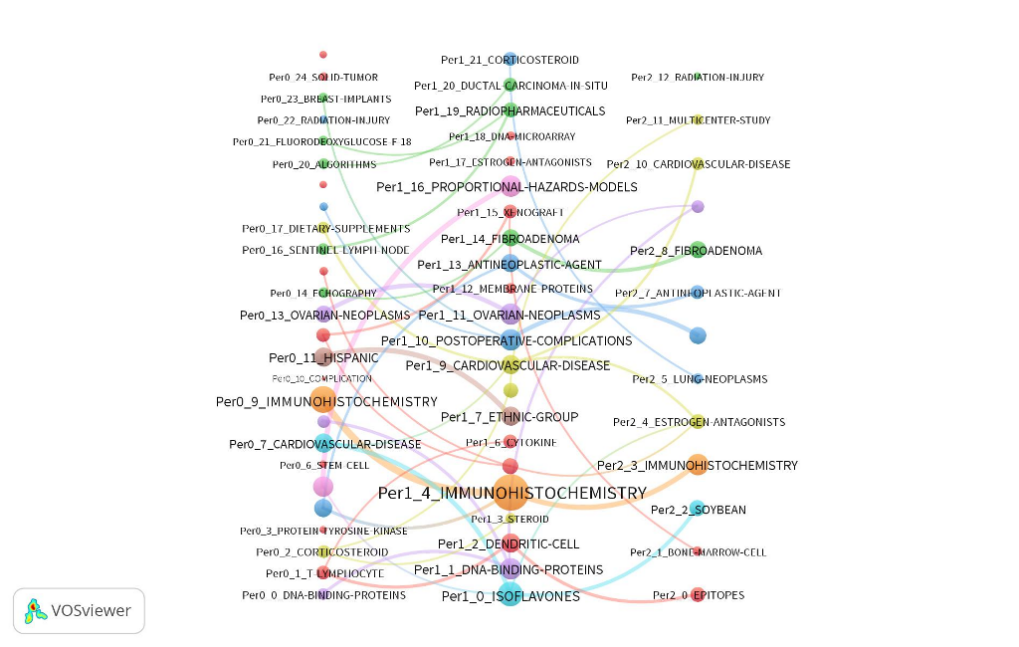
Thematic trends in the field of breast cancer from 1988 to 2020. Date adopted from global statistics retrieved from the Web of Sciences.

As in this scientometric evaluation of breast cancer topic, a very long period was considered for data collection. Moreover, due to the multiplicity of the publications, this assessment was divided into 3 decades from 1989 to 2020. The results of this study confirmed the progression of studies in recent decades and different concentrations of assessments completed in different years. Moreover, communications between these themes were also shown (Figure 8).

### Emerging Themes from the Three Evaluation Periods

Using SciMAT [22] and Vos-viewer [45], the research output in the field was observed to revolve around 8 areas. As shown in Multimedia Appendix 3, the themes in the rightmost column included *radiation injury, cardiovascular disease, fibroadenoma, antineoplastic agent, estrogen antagonistic, immunohistochemistry, soybean,* and *epitopes*, as indicated with different colors. Thematic links are demonstrated by a solid line. The size of these nodes is proportionate to the number of documents under each theme. In addition, the color of the nodes indicates different areas.

As seen in Figure 4, the analyzed research output is categorized by solid cohesion. Most of the identified topics have been gathered via thematic nodes. They arise from a topic appearing in the previous period and show a continuous evolution with almost no jumps or gaps.

Regarding the starting period, thematic areas started in the first period (Figure 5, Figure 7, right panel). Thus, they can be considered as the primary subjects in breast cancer. Furthermore, in the second period, a new thematic area emerged: *ethnic group, proportional hazards, corticosteroids, postoperative, ovarian neoplasm, ethnic group,* and *cytokine.* Indeed, the emerged thematic areas play an essential role in the development of the field. Regarding the theme composition, the thematic areas of *immunohistochemistry* are mainly composed of motor themes across all three periods. Furthermore, in the third period, *ethnicity* evolved to *Hispanic*. In addition, topics such as stem cell, solid tumor, breast implant, echography, and protein tyrosine kinase emerged in this field with some of them evolving from the second period.

The relationship between IHC and cancer biology, which is now better known, has influenced axillary lymph node dissection (ALND). Tumor biological factors are different in each tumor if tumor tend to metastasize to visceral or lymph nodes depends on tumor biological features. With advanced knowledge and understanding of tumor biology, systemic therapy and targeted therapy policies have changed. At present, the decision to initiate and prescribe chemotherapy (ie, systemic therapy) is influenced by the tumor stage and tumor biological factors, as well as the patient’s lymphatic status. For example, in some cases, a tumor is diagnosed by screening mammography in the early stages, and there is no lymphatic involvement. Decisions to continue adjuvant treatments depends on the biological factors of the tumor. Biological factors play a key role on the decision to start neoadjuvant therapy. For example, triple-negative, and Her2neu-positive tumors have a dermatological response to neoadjuvant therapy.

In patients with a positive sentinel lymph node biopsy (SLNB), tumor biological factors such as ER/PR/Her2neu are prognostic factors, and it is of interest to know whether ALND changes the patient’s cervix. According to the AMAROS trial [46], axillary radiotherapy was comparable to axillary dissection for local axillary control and even had fewer side effects. In patients with T1 and T2 masses, who had a positive SLNB and received axillary radiotherapy, overall survival and disease-free survival were similar to those who underwent axillary dissection. Therefore, SLNB is currently recommended for many patients with breast cancer. Currently, based on the IBCSG23-01 study, the National Comprehensive Cancer Network guidelines recommend only radiotherapy for patients with a positive SLNB (micrometastasis), without axillary dissection. Thus, SLNB has currently replaced ALND in many cases.

The false-negative rate is low in cases where a dual agent is used and at least more than two SLNs are found in patients with clinical lymph nodes (N1). Lymph node biopsy can be performed for patients undergoing neoadjuvant therapy. However, dual-agent therapy is preferably used when finding at least two lymph nodes in patients with preneoadjuvant clinical lymph node N1.

According to the AJCC (American Joint Committee on Cancer) staging, biomarkers such as ER/PR/Her2neu are recommended to be effective.

### Pathological Analysis

Currently, the basis of breast cancer treatment is complete knowledge of its progression and biological factors (ER/PR/Her2neu). These factors affect the stage of the disease and also indicate the likelihood of tumor recurrence. They can also assist in response to selected treatments.

### Conclusion

Eventually, scientometric analysis can showcase the current state of the science. Similarly, co-word analysis determines the frequency of words and thus indicates the most important research topics of a field. Using these methods, the characteristics and challenges of research fields and scientific disciplines can be determined. In addition, scientometric analysis of breast cancer research can be regarded as a roadmap for future research and policymaking in this important field of study.

## Appendix

Multimedia Appendix 1 Strategic diagram for the second evaluation period (1999 to 2009).

Multimedia Appendix 2 Strategic diagram for the second evaluation period (1999 to 2009).

Multimedia Appendix 3 Strategic diagram for the third evaluation period (2010 to 2020).

## Abbreviations

AJCC: American Joint Committee on Cancer
ALND: axillary lymph node dissection
IHC: immunohistochemistry
JAMA: Journal of American Medical Association
MeSH: Medical Subject Headings
NLM: National Library of Medicine
SLNB: sentinel lymph node biopsy
RIS: research information system
