# Supplementary figures and images for: Emerging Trends and Thematic Evolution of Breast Cancer: Knowledge Mapping and Co-Word Analysis
Source: JMIR Cancer. 2021 Oct 28;7(4):e26691. doi: 10.2196/26691 (PMC8587182; doi:10.2196/26691)

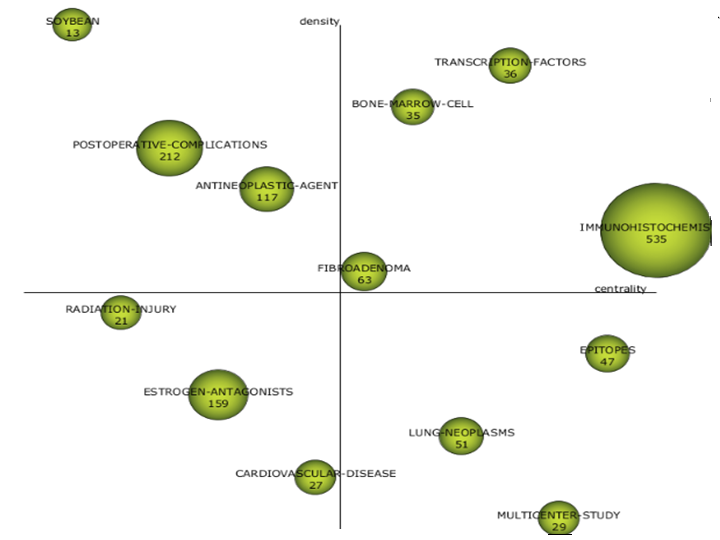

Supplement: Multimedia Appendix 1 [file cancer_v7i4e26691_app1.png]

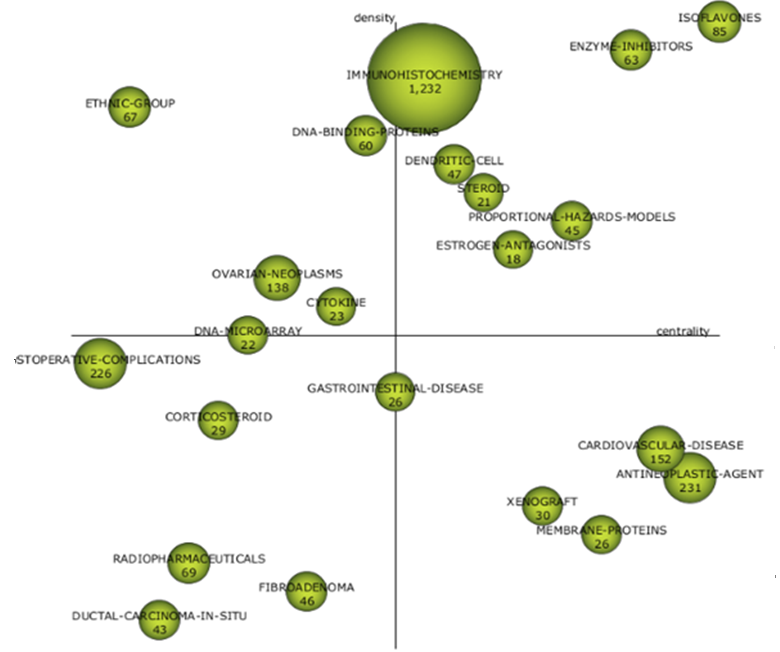

Supplement: Multimedia Appendix 2 [file cancer_v7i4e26691_app2.png]

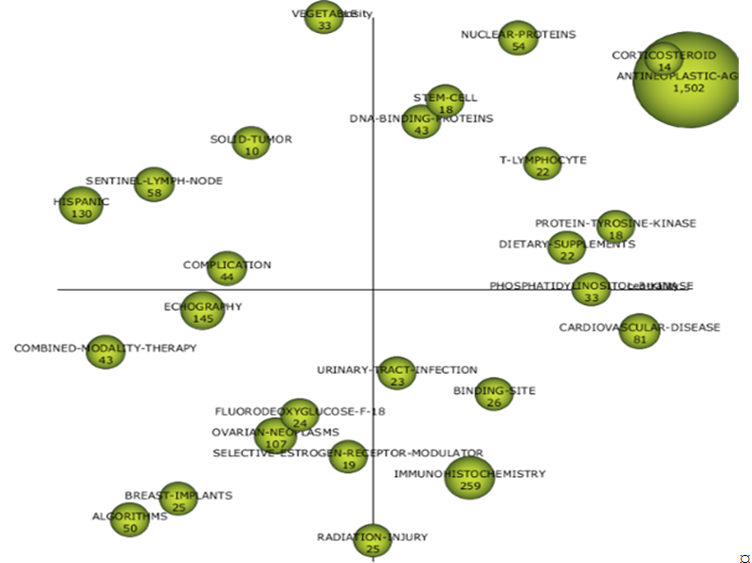

Supplement: Multimedia Appendix 3 [file cancer_v7i4e26691_app3.png]
